# Defects Detection Method Based on Programmable Spoof Surface Plasmon Polaritons in Non-Metallic Composites

**DOI:** 10.3390/mi14040756

**Published:** 2023-03-29

**Authors:** Jieping Wu, Xiaoqing Yang, Piqiang Su, Wenping Yu, Li Zheng

**Affiliations:** 1 College of Electronics and Information Engineering, Sichuan University, Chengdu 610065, China; 2 School of Automation and Electrical Engineering, Chengdu Technological University, Chengdu 611730, China

**Keywords:** spoof surface plasmon polaritons (SSPPs), split ring resonator (SRR), asymptotic frequency, defect detection method

## Abstract

Microwave nondestructive testing (NDT) offers promising application prospects due to its advantages of non-contact inspection in detecting defects in non-metallic composites. However, the detection sensitivity of this technology is generally affected by the lift-off effect. To reduce this effect and highly concentrate electromagnetic fields on defects, a defect detection method using scanning instead of moving sensors in the microwave frequency range was proposed. Additionally, a novel sensor based on the programmable spoof surface plasmon polaritons (SSPPs) was designed for non-destructive detection in non-metallic composites. The unit structure of the sensor was made up of a metallic strip and a split ring resonator (SRR). A varactor diode was loaded between the inner and outer rings of the SRR, and by changing the capacitance of this diode using electronic scanning, the field concentration phenomenon of the SSPPs sensor can be moved along a specific direction for defect detection. By using this proposed method and sensor, the location of a defect can be analyzed without moving the sensor. The experimental results demonstrated that the proposed method and designed SSPPs sensor can be effectively applied in detecting defects in non-metallic materials.

## 1. Introduction

Non-metallic composites are a type of material composed of two or more constituent materials. These constituents are carefully selected to create a material with unique properties that cannot be produced by any single material on its own. One of the most significant benefits of non-metallic composites is their ease of processing. They can be easily molded into different shapes and sizes without losing their structural integrity. This makes them an ideal choice for industries that require complex and intricate designs. Moreover, non-metallic composites have high strength, which means that they can withstand extreme conditions without breaking or deforming. This property makes them useful in applications that require materials to withstand high stress and pressure. Another advantage of non-metallic composites is their light weight. They are much lighter than traditional metallic materials, which makes them an excellent choice for industries that require lightweight yet strong materials. For example, the lightweight nature of non-metallic composite materials makes them ideal for use in aircraft, where weight savings can lead to significant fuel savings and improved performance. Thus, non-metallic composites are often preferred in industries that require high strength and low weight, such as aerospace and automotive industries [1,2].

However, despite these benefits, non-metallic composites face challenges in terms of their internal structure, which can be prone to defects after long-term use. Fatigue accumulation, corrosion, and other factors can all contribute to these issues. Additionally, the benefits of non-metallic composites can be quickly diminished if the internal structure of materials is compromised. Therefore, studying effective methods for evaluating the quality of non-metallic composites can minimize potential safety hazards and ensure that these materials continue to be used effectively in industry. In conclusion, non-metallic composites offer many advantages in aerospace applications. However, it is crucial to study effective methods for evaluating their quality to avoid potential safety hazards. By doing so, we can continue to rely on these materials for their performance and flexibility benefits while ensuring the safety [1].

The commonly used methods for detecting defects in non-metallic composites are ultrasonic testing, radiographic testing, infrared testing, and microwave nondestructive testing [3,4,5,6,7]. Ultrasonic testing requires a coupling medium to contact the object being tested, which may cause damage to it, and this can be a limitation in certain situations. Radiographic testing has advantages in detection accuracy, but it affects the health of operators. Infrared testing is sensitive to changes in ambient temperature, which can affect the accuracy of the results. Microwave nondestructive testing, which utilizes the interaction principle of microwave and matter, is an effective way of detecting defects by analyzing the changes of microwave parameters. In this method, a vector network analyzer is commonly used, producing low output power with most of the microwave energy acting on the object being tested, making it a safer option for operators [8]. Microwave non-destructive testing is widely studied by scholars due to its advantages of non-contact detection, easy integration, and high robustness [6,7,8,9]. The non-contact nature of this method makes it suitable for testing delicate objects that cannot withstand physical contact. Additionally, it can be easily integrated into existing testing systems, making it a cost-effective solution. Its high robustness also ensures that it can detect defects with high accuracy, even in challenging environments. In summary, while the various methods of defect detection in non-metallic composites have their advantages and disadvantages, microwave nondestructive testing stands out due to its non-contact nature, easy integration, and high robustness. As such, it is a popular choice among scholars and manufacturers alike for ensuring the quality and reliability of non-metallic composites.

In recent years, many types of microwave nondestructive testing sensors were designed to detect defects [7,10], such as horn antenna sensors, waveguide sensors, split ring resonators (SRR) sensors, and comprehensive split ring resonator (CSRR) sensors, etc. However, the volume of antenna sensor and waveguide sensor is generally large, and their detection sensitivity is limited. SRR structures were first announced by Pendry J et al., in 1999 [11]; they can highly concentrate electromagnetic fields in a small area and make the resonator sensitive to changes in the surrounding environment. Therefore, many sensors based on SRR were studied [12,13,14,15]. Among the many modified and derived structures of SRR, the CSRR is the most common [16,17]. Comparing CSRR with SRR, SRR is more suitable for miniaturized detection areas as a sensor [12,13,14,18], but their detection sensitivity is affected by lift-off effect, and the detection process requires frequent moving over the object to be tested.

Surface plasmon polaritons (SPPs) attracted the attention of scholars due to their strong binding ability to the electromagnetic fields. SPPs are surface electromagnetic waves generated by the coupling of external electromagnetic fields and metal electrons under specific conditions [19]. However, surface electromagnetic waves cannot be transmitted in microwave bands. To overcome this problem, spoof surface plasmon polaritons (SSPPs) are proposed, and then, surface electromagnetic waves can be excited by specific periodic structure in microwave wave bands. The dispersion characteristics of SSPPs can be determined by the geometry of unit structures [20]. Compared with the traditional transmission line structures, SSPPs has advantages of anti-interference, miniaturization, low crosstalk, etc. Therefore, SSPPs became a research hotspot in the microwave field [21,22,23], such as permittivity measurement [24], concentration measurement [25], defect detection [26], etc. However, the stability of these methods is affected.

The emergence of new concepts in reconfigurable metamaterials led to the proposal of digitally encoded metamaterials as a means of dynamically controlling electromagnetic waves. This was achieved by designing different cell structures as digital bits (“0” or “1”). Building upon this concept, Cui T. et al. [27] proposed that the lumped resistance or capacitance in a microstrip circuit can be equivalent to a material with a specific conductivity or permittivity. Thus, SSPPs transmission paths can be controlled by changing the parameters of electronic components. This idea points towards a new direction of designing programmable SSPPs sensors to control the direction of field concentration phenomenon, rather than moving the sensors. Such an approach can be beneficial in reducing the lift-off effect.

To achieve high-precision detection for microwave non-destructive testing, the electromagnetic fields should be highly concentrated at the defect of test object while reducing the lift-off effect. This paper presents the design of a programmable SSPPs sensor based on the concept of digital coding metamaterials on SSPPs, where each unit structure is composed of a metallic strip and SRR. The rest of this paper is organized as follows. Section 2 introduces the theory and structure of the SSPPs sensor. Section 3 elaborates and analyzes the experiment process and results. Finally, Section 4 provides a conclusion.

## 2. Theory and Structure of SSPPS Sensor

Controlling the propagation direction of electromagnetic fields on a sensor is a key problem in defect detection. The dispersion property $kd_{SSPPs}\;$of SSPPs structure is often used to study the propagation property of electromagnetic fields on SSPPs structure. The property is controlled by the dimensions of the SSPPs unit structure, and can be expressed as follows [28]:

(1) $$kd_{SSPPs}=k_{0}\sqrt{d^{2}+a^{2}\tan^{2}\left(k_{0}h\right)}$$

Here,$\;k_{0}$ is the wavenumber in free space, and it has a close relationship with the material permittivity of electronic components. The parameters a, d, and h are the dimensions to describe the structure. When the value of $kd_{SSPPs}\;$reaches 180 degrees, the electromagnetic fields of the SSPPs structure are bound in a certain area, and they lose the ability to transmit forward. Under these circumstances, the corresponding frequency is called the asymptotic frequency [11].

In practical engineering, it is inconvenient to control the asymptotic frequency by changing the material permittivity. Instead, it is proposed to change the parameters of electronic components, which is equivalent to changing the material permittivity. Based on this concept, a sensor with a programmable SSPPs structure for defect detection was proposed by adding tunable electronic components, such as varactor diodes, variable inductor, graphene, etc.

**Figure 1:**
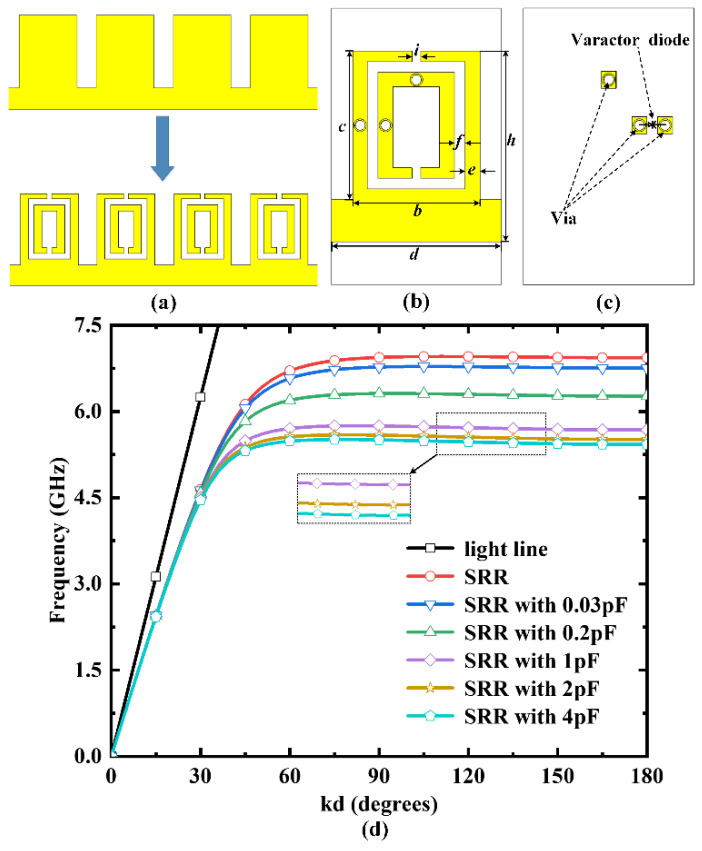
The unit structure of the programmable SRR and dispersion curves. (**a**) Evolution of the SRR structure. (**b**) The top view. (**c**) The bottom view. (**d**) The dispersion curves.

The SRR structure is nested on SSPPs to form a new kind of SSPPs sensor. Here, varactor diodes were used as an example to analyze the control on the asymptotic frequency of the SRR structure, and were also used as a device to change the capacitance value by adjusting the bias voltage. The unit structure of SRR is shown in Figure 1a, with the following dimensions: *b* = 3 mm, *c* = 3.5 mm, *d* = 4 mm, *e* = 0.35 mm, *f* = 0.25 mm, *h* = 5.5 mm, *i* = 0.25 mm. The thickness of copper was 0.035 mm. The corrugated structure composed of ultra-thin metal materials evolved into a periodic SRR array loaded on one side of the metal strip. The bottom view and top view of the unit structure are shown in Figure 1b and Figure 1c, respectively. On the bottom view, the varactor diode was loaded on the substrate, which was connected to the inner and outer rings of the SRR by metallic vias. The dielectric substrate material was F4B (relative permittivity 2.65, loss tangent 0.002), and the thickness was 1 mm.

The dispersion curves at different capacitance, based on the fundamental mode of the proposed SSPPs unit structure, can be used to analyze the relationship between frequency and *kd* degrees. Here, a SRR structure with nothing loaded and SRR structure with different capacitance loaded were selected for dispersion analysis. The experiment results can be seen in Figure 1d, where the black line stands for the light line in free space, and the figure clearly shows that the asymptotic frequency was higher when there was a dielectric plate loaded varactor diode. Meanwhile, the asymptotic frequency decreased with the increase in the capacitance of the varactor diode. Hence, the capacitance can be changed by programming the loaded varactor diode, which can control the dispersion property of SRR structure reasonably and realize reconfigurable SSPPs unit structure.

**Figure 2:**
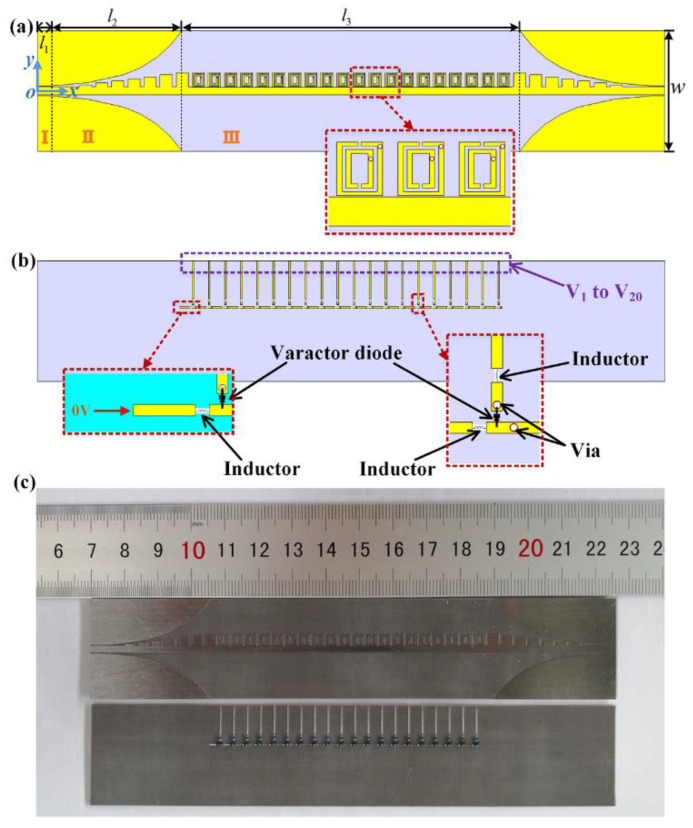
The schematic of the period SSPPs structure (**a**) The top view. (**b**) The bottom view. (**c**) The fabricated SSPP structure.

Based on the dispersion property of the proposed SSPPs unit structure, a new kind of SSPPs sensor was designed, as shown in Figure 2. The top view of the SSPPs sensor consisted of three parts, as seen in Figure 2a. Part I shows the co-planar waveguide. Part II shows the gradient structure and the flaring ground. Part III shows N-cascaded SSPPs unit structure (*N* = 20) with the following dimensions: *l*_1_ = 3 mm, *l*_2_ = 33 mm, *l*_3_ = 84 mm, and *w* = 30 mm. In the figure, the yellow part represents copper, and the other part represents F4B.

The bottom of SSPPs Sensor is shown in Figure 2b, which was composed of varactor diodes and inductors. To avoid the friction damage to sensor during the testing process, the varactor diodes were placed on the back of the substrate. The SRR unit loaded on SSPPs sensor at different location can be selected by controlling the varactor diodes. Furthermore, a high-impedance line was designed to better load the DC bias voltages, which aimed at controlling the capacitance of the varactor diodes and relating to the outer rings of SRR through vias.

Figure 2c shows the fabricated SSPPs device based on the standard PCB fabrication process. To prevent signal crosstalk of DC bias voltages and radio frequency, a metal wire was loaded onto the back of the substrate, which relates to the inner rings of SRR through vias. The inductors with 30 nH were loaded in lines to eliminate the mutual interference between signals. The metal wire on the back of the substrate was loaded with 0 V voltage, and the high-impedance lines were loaded with DC bias voltages. The capacitance of the varactor diode could be controlled independently by programming the bias voltages on each line, which ranged from V_1_ to V_N_, respectively. Meanwhile, the SRR unit loaded on the SSPPs sensor at different locations could be selected for defect detection; this process is called dynamic control, also known as the electronic scanning method.

## 3. Experiment Results and Discussion

### 3.1. Experiment

In order to assess the potential of the proposed SSPPS sensor for detecting defects, a series of experiments were carried out. These experiments focused on controlling the location of the field concentration phenomenon and detecting defects in non-metallic materials by utilizing the electric scanning method.

To verify the feasibility of controlling the location of the field concentration phenomenon, an experiment was conducted using the proposed SSPPS sensor. Twenty SSPPs units were used and divided into two groups. The first group consisted of a single unit loaded with capacitance ($C_{v1}$), while the second group consisted of the remaining 19 units loaded with a different capacitance ($C_{v2}$). According to the dispersion curves of programmable SSPPs units, the sensor has two rejection bands if the capacitance difference ($∆C_{v}=\left|C_{v1}-C_{v2}\right|$) between the two groups is large enough. Therefore, by programming to change the capacitance of the varactor diode, it is possible to move and control the location of field concentration phenomenon of SSPPs. For this experiment, the 10th SSPPs unit was selected as the first group with capacitance ($C_{v1}$), which had a value of 2.1 pF. The remaining 19 SSPPs units were selected as the second group loaded with the different capacitance $C_{v2}$, which were set to 0.23 pF. Then, the transmission coefficients ($S_{21}$) of the sensor were analyzed, and the field concentration phenomenon of the SSPPs sensor at different frequency near the rejection bands was also discussed. Next, the field concentration phenomenon of the SSPPs sensor was further analyzed by programming to change the capacitance of the varactor diode. For this, the capacitor value ($C_{v1}$) was set to “1”, while the unit for capacitor value ($C_{v2}$) was set to “0”. Finally, the moving process of the field concentration phenomenon of the SSPPs sensor was verified with electronic scanning method. The main content of the experiment was the “1” is sequentially moved in one direction by programming the external bias voltages.

To verify the feasibility of the proposed method, a Rogers 4350B board was selected as the test sample, as it is a widely used non-metallic composites with a relative permittivity of 3.66, a loss tangent of 0.037, and a thickness of 2 mm. A sample contained three defects, which were cylinders with different radii but the same height (1 mm). The defects were located in the fifth and sixth, 13th, and 18th SSPPs units. Additionally, another sample had no defects with the same material. As the main structure of the sensor was SRR, changing the size of the SRR structure can alter the resonance frequency. Additionally, the presence of a defect below the SRR structures can cause a change in the capacitance value of the SRR, leading to a shift in the resonance frequency. Therefore, the shift in resonance frequency was the primary indicator for evaluating non-metallic composites using our proposed method. Thus, we repeated the process of moving the field concentration phenomenon of the SSPPs sensor with electronic scanning on the test samples. The bias voltages were programmed to move “1” along a direction, and the obtained resonance frequency was used for evaluation.

### 3.2. Results and Discussion

**Figure 3:**
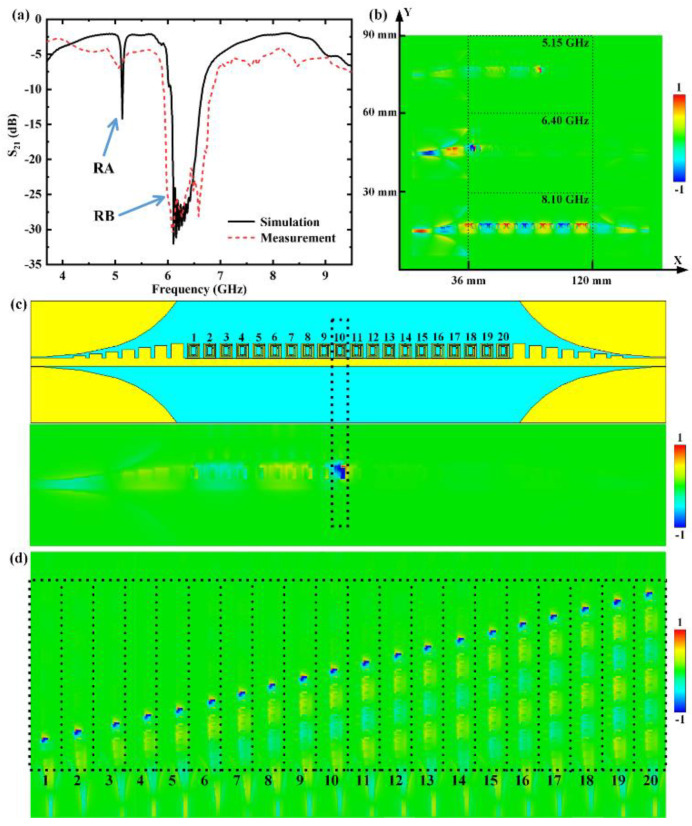
Results of the SSPPs sensor when all the units are divided into two groups. (**a**) The simulated and measured transmission coefficient of the SSPPs sensor. (**b**) The simulated normalized z-component electric field distributions at 5.15 GHz, 6.40 GHz and 8.10 GHz, respectively. (**c**) The simulated normalized z−component electric field distributions of the tunable SSPPs sensor at 5.15 GHz, and where the 10th unit is “1” and other units are “0”. (**d**) The process of electromagnetic waves transmission control with electronic scanning.

The simulated and measured transmission coefficients of the SSPPs sensor can be seen in Figure 3a, when $C_{v1}=2.1\;\mathrm{pF}$ and $C_{v2}=0.23\;\mathrm{pF}$. Due to a large capacitance difference $∆C_{v}$, there were frequency bands of the two rejections in simulation and measurement, called RA and RB. Rejection RA occurred at 5.15 GHz, and RB occurred at 6.10–6.70 GHz.

As can be seen from Figure 1d, when the SSR capacitance value was 2 pF, the cutoff frequency was about 5.1 GHz. When the SSR capacitance was 0.2 pF, the cutoff frequency was around 6.1 GHz. Thus, the formation of rejection RA was controlled by the 10th SSPPs unit loaded with a capacitance ($C_{v1}$) of 2 pF, while the formation of rejection RB was introduced by the remaining 19 SSPPs units loaded with another capacitance ($C_{v2}$) of 0.23 pF together. Because the rejection RA had sharper peaks, the frequency of the rejection RA was considered the resonant frequency of the SSPPs sensor. The results indicate that we can regulate the resonant frequency of the sensor by setting different capacitance values. The transmission coefficient measured had a large attenuation at the resonant frequency, which was due to the large insertion loss of the SSPPs sensor loaded with many additional electronic components. 

Figure 3b displays the normalized field concentration phenomenon of the SSPPs sensor at 5.15 GHz, 6.40 GHz, and 8.10 GHz, respectively. At 5.15 GHz (rejection RA), the field concentration phenomenon was at a specific location in region 3 and no longer transmitted forward due to the resonant frequency of the sensor. At rejection RB (6.4 GHz), the electric field could not continue to be transmitted after reaching the head of region 3. At 8.10 GHz, electromagnetic waves could be uniformly transmitted in SSPPs devices without interference from the two rejections. When the working frequency was 5.5 Hz, the SSPPs sensor with a periodic SRR structure had an advantage in increasing magnetic flux density and concentrating the electric field energy, compared with the SSPPs sensor with comb structure (without a SRR periodic structure) [11]. This phenomenon is particularly beneficial for local defect detection.

As depicted in Figure 3c, a field concentration phenomenon occurred at the 10th SSPPs unit structure of the SSPPs sensor, where the unit was loaded with $C_{v1}$, and the remaining 19 units were loaded with $C_{v2}$. The tunable SSPPs sensor employed the concept of electronic scanning, which enabled the controlled movement of the field concentration phenomenon by programming. The capacitor value of $C_{v1}$ was set at “1”, while the capacitor value of $C_{v2}$ was set at “0”. This means only one of the 20 units in a row had a value of “1”. By programming the external bias voltages, the “1” was sequentially moved in a single direction. As shown in Figure 3d, the field concentration phenomenon also moves in this direction. Thus, the electronic scanning method can be leveraged to control the location of field concentration phenomenon of SSPPs sensor.

**Figure 4:**
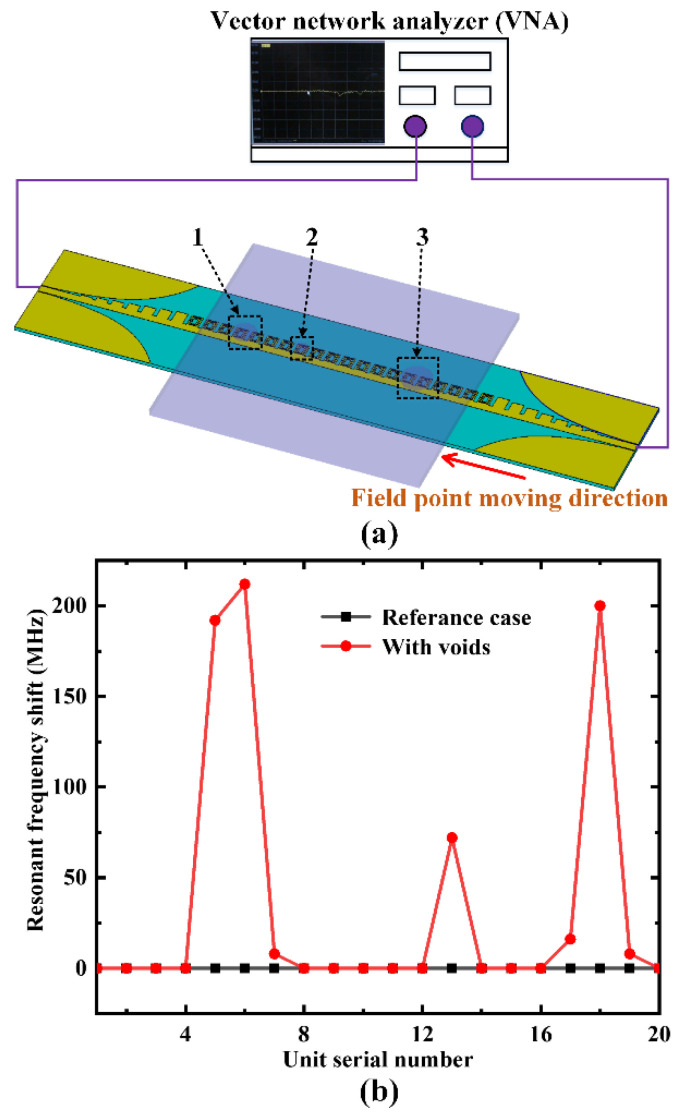
The experiment to verify the electronic scanning process for defect detection. (**a**) The scanning schematic of SSPPs sensor, where “1~3” are the defects located in the test sample. (**b**) Experimental results for resonant frequency shift as a function of unit position for defects.

Figure 4a shows a Rogers 4350B board (dielectric constant = 3.66, loss tangent = 0.037) with three different cylindrical defects: defect 1, defect 2, and defect 3. The defects had the same height but different radii. Among them, defect 3 had the largest volume and defect 2 had the smallest. The proposed SSPPs sensor worked at the resonant frequency of 5.15 GHZ. As the field concentration phenomenon moved to the defects of the test sample, the resonant frequency of 5.15 GHZ shifted. Bias voltages were programmed to move “1” along the direction of the arrow shown in Figure 3a.

Figure 4b shows the change in resonance frequency shift as the field concentration phenomenon moves from unit “1” to unit “20” in sequence. Additionally, there were obvious resonance frequency shifts at the fifth, sixth, thirteenth, and eighteenth SRR units, indicating that there were some defects above the 4 SRR units, corresponding to the “1”, “2”, and “3” defects in Figure 4a. Because defect 3 had the largest volume, which had the greatest impact on the electric field distribution, its resonant frequency shift had the maximum value. On the contrary, defect 2 had the smallest volume and had a small impact on the electric field distribution. Its resonant frequency shift was the minimum value. Defect 3 had the largest volume and, therefore, had the greatest impact on the electric field distribution. As a result, it had the maximum resonant frequency shift. On the other hand, defect 2 had the smallest volume and had a small impact on the electric field distribution. Consequently, its frequency shift was the minimum value.

Based on the experimental results, it can be concluded that that the resonant frequency of the sensor can be regulated by setting different capacitance values, and the location of field concentration phenomenon can be controlled by electronic scanning method. When the field concentration phenomenon moved in one direction on the test object, a resonant frequency shift phenomenon can indicate the presence of defects in the non-metallic material located in the area. This method not only reduced the lift-off effect caused by frequent mechanical movements, but also used electronic scanning to expand the detection area. This helped to reduce the detection blind area and ultimately improve detection efficiency. The results of these experiments provided insight into the feasibility of the sensor for detecting defects in non-metallic materials.

## 4. Conclusions

Non-metallic composite materials are widely used in industry. Studying effective methods to evaluate their quality is essential for avoiding potential safety hazards. While various methods of defect detection in non-metallic composites have their advantages and disadvantages, microwave non-destructive testing stands out.

In this work, to study a method to improve the detection sensitivity of microwave non-destructive testing in non-metallic composites, the relationship between the parameters of electronic components and asymptotic frequency was analyzed according to the dispersion characteristics of SSPPs. Then, to avoid the lift-off effect and concentrate the electric field on defect, a novel sensor combining SRR and SSPPs was proposed based on programmable structure, and the electric scanning was utilized to determine the location of the defect rather than moving the sensor. For the sensor, the capacitance of varactor diodes in the SSPPs units were changed to realize the change of the SSPPs dispersion by programming the bias voltages. Based on the electromagnetic field distribution of SRR, the proposed SSPPs units could be programmed in groups to achieve a concentration of the electromagnetic field intensity. The experimental results showed that the electronic scanning method can be used to control the location of field concentration phenomenon of the SSPPs sensor instead of moving sensor, and the proposed method and the SSPPs sensor can be applied in defects detection in non-metallic composites. This is helpful in improving the detection sensitivity in the field of microwave non-destructive testing.

The SSPPs are programmable, which allows for greater flexibility in the design of the sensor, thereby providing new possibilities for the improvement of detection capabilities. Additionally, it has the potential to greatly improve the accuracy of defect detection in non-metallic composites, which is important for many industries. Thus, this research has a certain contribution to Micromachines. The following works will continue to be carried out:

(1) The proposed method and sensor can achieve preliminary localization of defects, but the size and shape of defects cannot be evaluated. In the future, it is mainly considered to introduce neural network learning algorithms to train and test microwave parameters.
(2) Due to the addition of simple circuits, the proposed SSPPs sensor loses its flexible characteristics, making it difficult to detect defects in irregular objects. In the future, we will mainly consider using new materials to control the concentration of the electric field instead of the electrical scanning method.

## Data Availability

The data that support the findings of this study are available from the corresponding author upon reasonable request.
